# Multiple Roles of 1,4-Diazabicyclo[2.2.2]octane in the Solvothermal Synthesis of Iodobismuthates

**DOI:** 10.1021/acs.inorgchem.1c00318

**Published:** 2021-03-22

**Authors:** Yunhe Cai, Ann M. Chippindale, Richard J. Curry, Paz Vaqueiro

**Affiliations:** † Department of Chemistry, University of Reading, Whiteknights, Reading, Berkshire RG6 6DX, United Kingdom; ‡ Photon Science Institute, Department of Electrical and Electronic Engineering, University of Manchester, Manchester, M13 9PL, United Kingdom

## Abstract

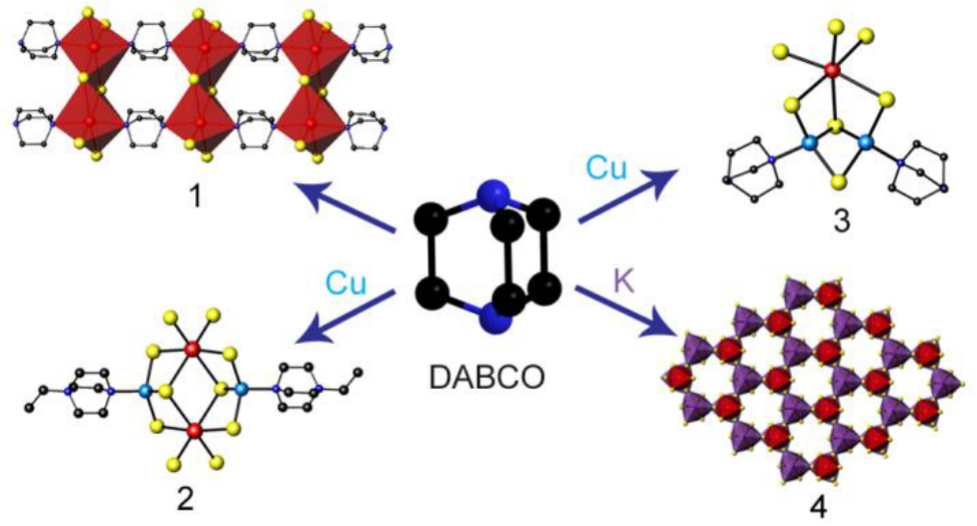

Hybrid bismuth-containing halides are emerging as alternative candidates to lead-containing perovskites for light-harvesting applications, as Bi^3^^+^ is isoelectronic with Pb^2^^+^ and the presence of an active lone pair of electrons is expected to result in outstanding charge-carrier transport properties. Here, we report a family of one binary and three ternary iodobismuthates containing 1,4-diazabicyclo[2.2.2]octane (DABCO). These materials have been prepared solvothermally and their crystal structures, thermal stability, and optical properties determined. Reactions carried out in the presence of bismuth iodide and DABCO produced (C_6_H_12_N_2_)BiI_3_ (**1**), which consists of hybrid ribbons in which pairs of edge-sharing bismuth octahedra are linked by DABCO ligands. Short I···I contacts give rise to a three-dimensional network. Similar reactions in the presence of copper iodide produced (C_8_H_17_N_2_)_2_Bi_2_Cu_2_I_10_**(2)** and [(C_6_H_13_N_2_)_2_BiCu_2_I_7_](C_2_H_5_OH) **(3)** in which either ethylated DABCO cations (EtDABCO)^+^ or monoprotonated DABCO cations (DABCOH)^+^ are coordinated to copper in discrete tetranuclear and trinuclear clusters, respectively. In the presence of potassium iodide, a unique three-dimensional framework, (C_6_H_14_N_2_)[(C_6_H_12_N_2_)KBiI_6_] **(4)**, was formed, which contains one-dimensional hexagonal channels approximately 6 Å in diameter. The optical band gaps of these materials, which are semiconductors, range between 1.82 and 2.27 eV, with the lowest values found for the copper-containing discrete clusters. Preliminary results on the preparation of thin films are presented.

## Abstract 2

The solvothermal syntheses, crystal structures, and optical properties of four new iodobismuthates containing 1,4-diazabicyclo[2.2.2]octane (DABCO) are described. In the absence of hydriodic acid, DABCO can act as a ligand instead of acting as a countercation.

## Introduction

Lead and bismuth halides have long been investigated due to their optical and electronic properties.^1^ Interest in these materials has grown exponentially since the discovery in 2009 of the potential use of methylammonium lead triiodide (MAPI) as a photovoltaic material,^2^ which has been incorporated into solar cells with conversion efficiencies exceeding 20%.^3^ The exploitation of MAPI in commercial solar cells may however be limited by the toxicity of lead and the intrinsic instability of these materials.^4^ As the presence of an active lone pair of electrons in Pb^2+^ is believed to be key for the exceptional properties of lead-containing halides,^5^ the search for alternative materials is focused on halides containing post-transition-metal cations with an n*s*^2^ electronic configuration. Of these, Bi^3+^ is particularly attractive, as it is isoelectronic with Pb^2+^ and its toxicity is low.

The structural chemistry of iodobismuthates is however markedly different from that of iodoplumbates. In particular, iodobismuthates exhibit a great deal of structural diversity: polymeric, discrete polynuclear, or mononuclear anionic units have been found, depending upon the countercations and synthetic conditions used.^6^ Dinuclear anions, such as [Bi_2_I_8_]^2–^, [Bi_2_I_9_]^3–^, and [Bi_2_I_10_]^4–^, are particularly common,^7^ although polynuclear anions, such as trinuclear (e.g., [Bi_3_I_11_]^2–^),^7b^ tetranuclear (e.g., [Bi_4_I_16_]^4–^),^8^ and up to octanuclear units, e.g., [Bi_8_I_28_]^4–^,^9^ are also known. In these iodobismuthates, the Bi^3+^ cation usually adopts a distorted octahedral coordination, existing as either discrete anions or as polynuclear anions containing edge- or face-sharing octahedra. A number of one-dimensional polymeric units are known, such as edge-sharing [BiI_4_]^−^ chains,^10^ while structures of higher dimensionalities are extremely rare, and examples appear to be limited to a 2-dimensional metal-deficient perovskite, (H_2_AEQT)Bi_2/3_I_4_ (AEQT = 5,5′′′′-bis(aminoethyl)-2,2′:5′,2′′:5′′,2′′′-quaterthiophene),^11^ the layered A_3_Bi_2_I_9_ (A = K, Rb) phases,^12^ and (Me_2_C = NMe_2_)Bi_2_I_7_.^13^ One approach adopted to increase the dimensionality of iodobismuthates to produce lead-free materials that are structurally related to the photovoltaic lead perovskites is the heterovalent substitution of Pb^2+^ by Bi^3+^ together with the incorporation of a monovalent cation, such as Ag^+^ or Cu^+^. This has been successfully demonstrated with Ag^+^ in the double perovskites A_2_AgBiX_6_ (A = Cs, CH_3_NH_3_; X = Cl, Br, I)^14^ and [AE2T]_2_AgBiI_8_ (AE2T = 5,5′-diylbis(aminoethyl)-[2,2′-bithiophene]).^15^ In contrast, examples that incorporate Cu^+^ to form hybrid copper iodobismuthates are to date limited to a small number of discrete clusters,^16^ the one-dimensional chains [Cu_2_Bi_2_I_10_]^2–^ and [CuBi_5_I_19_]^3–^,^16b,17^ and the recently reported [CuBiI_8_]^4–^ layers.^18^

Here, we describe new hybrid iodobismuthates and copper iodobismuthates containing 1,4-diazabicyclo[2.2.2]octane (DABCO), an amine which can potentially act as a ditopic linker between two metal centers. In (C_6_H_12_N_2_)BiI_3_ (**1**), unprotonated DABCO acts as a linker between bismuth octahedra, while in (C_8_H_17_N_2_)_2_Bi_2_Cu_2_I_10_ (**2**) and [(C_6_H_13_N_2_)_2_BiCu_2_I_7_](C_2_H_5_O) (**3**), DABCO is coordinated to copper cations, and in (C_6_H_14_N_2_)(C_6_H_12_N_2_)KBiI_6_ (**4**), which is a remarkable example of a three-dimensional iodobismuthate, (DABCOH_2_)^2+^ acts as a countercation, while unprotonated DABCO molecules coordinate to potassium cations.

## Experimental Section

All compounds were synthesized in 23 mL Teflon-lined stainless steel autoclaves. Ethanol (>99.8%), ethylene glycol (99.8%), BiI_3_ (99%), CuI (98%), KI (≥99%), and DABCO (≥99%) were obtained from Sigma-Aldrich and used without further purification. In each of the reactions described below, the reagents were loaded into a Teflon liner and stirred for approximately 10 min, prior to the reaction vessel being sealed and heated. After being cooled to room temperature, the products were collected by vacuum filtration, washed with deionized water, and allowed to dry in air. Reactions were carried out between 130 and 170 °C with different molar ratios of reagents and varying volumes of solvent. A constant reaction time of 5 days was used, based on prior experience. The initial reaction conditions that resulted in the preparation of compounds **1**–**4** are described in the SI. The optimized reaction conditions for the preparation of large amounts of crystals of each compound, are described below. The purity of the products as synthesized was assessed using powder X-ray diffraction. Hand-picked crystals of **1**–**4** were characterized using elemental analysis (carried out by MEDAC LTD), single-crystal and powder X-ray diffraction, thermogravimetric analysis, and IR and UV–vis spectroscopy, as described below.

### Synthesis of (C_6_H_12_N_2_)BiI_3_ (1)

A mixture of BiI_3_ (0.5850 g, 1 mmol), 1,4-diazabicyclo[2.2.2]octane (DABCO, 0.0831 g, 0.75 mmol), and ethylene glycol (10 mL) was placed in an autoclave, which was heated to 140 °C for 5 days. The heating and cooling rates were 0.67 °C min^–1^. The solid product contained a large amount of red crystals, together with traces of an impurity in the form of an unidentified red powder. The red crystals (yield ≈ 70%) were identified as **1** by single crystal X-ray diffraction. Elemental analysis of **1**: C = 10.03%, H = 1.74%, N = 3.88%; calc: C = 10.27%, H = 1.72%, N = 3.99%.

### Synthesis of (C_8_H_17_N_2_)_2_Bi_2_Cu_2_I_10_ (2)

This compound was synthesized using BiI_3_ (0.5949 g, 1 mmol), CuI (0.1924 g, 1 mmol), KI (0.3256 g, 2 mmol), DABCO (0.0831 g, 0.75 mmol), and ethanol (10 mL). The vessel was heated to 170 °C for 5 days, using a heating and cooling rate of 0.83 °C min^–1^. The solid product consisted of red crystals of **2** (yield ≈ 70%). Elemental analysis of **2**: C = 9.11%, H = 1.67%, N = 2.72%; calc: C = 9.17%, H = 1.63%, N = 2.67%.

### Synthesis of [(C_6_H_13_N_2_)_2_BiCu_2_I_7_](C_2_H_5_OH) (3)

A mixture of BiI_3_ (1.1798 g, 2 mmol), CuI (0.2858 g, 1 mmol), KI (0.3256 g, 2 mmol), DABCO (0.0831 g, 0.75 mmol), and ethanol (10 mL) was placed in a sealed autoclave, which was heated to 140 °C for 10 days. The heating and cooling rate was 0.67 °C min^–1^. The solid product consisted of red crystals of **3** (yield ≈ 60%), together with small amounts CuI and an unidentified impurity. Elemental analysis of **3**: C = 10.81%, H = 2.00%, N = 3.54%; calc: C = 11.23%, H = 2.15%, N = 3.74%.

### Synthesis of (C_6_H_14_N_2_)[(C_6_H_12_N_2_)KBiI_6_] (4)

This compound was prepared using a mixture of BiI_3_ (0.5860 g, 1 mmol), KI (0.4490 g, 3 mmol), DABCO (0.1145 g, 1 mmol), and ethanol (10 mL). The autoclave was heated to 140 °C for 5 days. The heating and cooling rates were 0.67 °C min^–1^. The product consisted of a mixture of a red powder, which is an unidentified impurity, and red crystals of **4** (yield ≈ 40%). Elemental analysis of **4**: C = 11.39%, H = 1.92%, N = 4.38%; calc: C = 11.65%, H = 2.10%, N = 4.53%.

### Single-Crystal Diffraction

Single-crystal X-ray diffraction data for crystals of **1**–**4** were collected using Mo Kα radiation (λ = 0.71073 Å) on either an Agilent Gemini S Ultra diffractometer (**1**–**3**) or a Rigaku XtaLAB Synergy diffractometer (**4**). A single crystal of each compound was mounted using Paratone-N oil and flash cooled to temperatures between 100 and 150 K under nitrogen in an Oxford Cryosystems Cryostream. Data reduction was carried out using the CrysAlisPro software.^19^ The structures were solved using Superflip^20^ and refined against *F* using the program CRYSTALS.^21^ All the hydrogen atoms were located in difference Fourier maps, then placed geometrically with a C–H distance of 0.95 Å and a *U*_iso_ of 1.2 times the value of *U*_eq_ of the parent C atom. The hydrogen atoms attached to C were then refined with riding constraints. All crystals from **3** were twinned. After repeated data collections to identify a suitable crystal, data were collected on a crystal from **3** consisting of two twins, related by a 180° rotation about the [001] reciprocal lattice direction. The organic ligand in **3** was modeled isotropically. The single-crystal data for compound **4** were treated with SQUEEZE^22^ to correct for the effect of the disordered organic moieties. Using a void probe radius of 1.2 Å, SQUEEZE found a void volume of 365 Å per unit cell, which contained 133 electrons. This is consistent with the presence of two DABCO moieties per unit cell (62 electrons each). The organic ligand in **4** was modeled isotropically. Selected crystallographic information is shown in Table 1. Data for compounds **1**–**4** have been deposited with the Cambridge Crystallographic Data Centre as CCDC 2020552–2020555, respectively.

**Table 1:** Crystallographic Data for Compounds **1–4**^a^

Compound	1	2	3	4
Crystallographic formula	C_6_H_12_N_2_BiI_3_	C_16_H_34_N_4_Bi_2_Cu_2_I_10_	C_14_H_31_N_4_OBiCu_2_I_7_	C_6_H_12_N_2_KBiI_6_
*M*_r_	701.87	2096.57	1495.83	1121.68^a^
Crystal habit	Red plate	Red plate	Red rod	Orange plate
Crystal system	Orthorhombic	Monoclinic	Monoclinic	Hexagonal
*T*/K	150	150	111	100
Space group	*F* m m m	*P* 2_1_/*n*	*P* 2_1_/*c*	*P* 6_3_*m c*
*a*/Å	7.7927(3)	8.9367(2)	8.9704(2)	10.16174(4)
*b*/Å	13.1065(5)	21.1327(5)	12.0354(3)	10.16174(4)
*c*/Å	24.0345(10)	10.8820(3)	29.1122(7)	14.79110(4)
α/°	90	90	90	90
β/°	90	96.361(2)	91.521(2)	90
γ/°	90	90	90	120
*Z*	8	2	4	2
ρ_cal_/g cm^–3^	3.798	3.409	3.162	2.816
*R*_merg_	0.031	0.036	0.108	0.085
*R*(*I* > 3.0σ(*I*))	0.0385	0.0373	0.1097	0.0456
*R*_w_	0.0530	0.0336	0.0982	0.0648
*GoF*	1.35	1.43	1.49	1.31

a Disordered DABCO not included.

### Characterization

The air-stable polycrystalline products as synthesized were characterized using a Bruker D8 Advance powder diffractometer, operating at room temperature with Ge-monochromated Cu Kα1 radiation (λ = 1.5406 Å) and a LynxEye linear detector. Data were collected over the angular range 5 ≤ 2θ/° ≤ 75. Le Bail refinements were carried out using TOPAS^23^ to determine the room-temperature lattice parameters (see SI) and establish the purity of the samples.

Further characterization measurements were made on hand-picked crystals of each compound. Thermogravimetric Analysis (TGA) was carried out using a TA-TGA Q50 instrument, operating under a flowing nitrogen atmosphere. Data were collected from room temperature to 750 °C, at a rate of 10 °C/min. Fourier Transform Infrared spectroscopy was carried out on ground samples using a PerkinElmer Spectrum 100 FT-IR spectrometer. UV–vis diffuse reflectance data were collected between 1100 and 200 nm using a PerkinElmer Lambda 35 UV–vis spectrometer. Each compound was finely ground and BaSO_4_ was used as a standard. The absorption data were calculated using the Kubelka–Munk function.^24^

Photoluminescence spectra were obtained under 405 nm (∼3 eV) excitation (∼300 mW/cm^2^) at room temperature using lock-in amplification. Emission was collected using optics and dispersed in a Bentham TMc300 monochromator using 1200 or 600 g/mm gratings and detected using Newport 818-SL or 818-IG calibrated detectors. All spectra have been corrected for the system response. A low-temperature photoluminescence spectrum of compound **2** was obtained using the same system with the sample cooled using an Oxford Instruments cryogen-free cryostat.

The solubility of each compound was assessed by placing a small amount of product (10–20 mg) in approximately 2 mL of either acetonitrile or DMF at room temperature. Thin films of those compounds found to be soluble were prepared by drop-casting a DMF solution onto a fluorinated tin oxide (FTO)-coated glass plate.

## Results

The initial reactions, which are described in the SI, produced mixtures of phases in the form of both powders and single crystals. Following structural characterization of the phases in single-crystal form, the stoichiometry of the reaction mixtures was adjusted to resemble those of compounds **1**–**4**. For compound **2**, this required inclusion of KI, as a source of iodide. In the initial reaction producing **1**, copper iodide was included, and it was subsequently found that its presence was not required for the synthesis of **1**. Changes in reaction temperature and in the volume of solvent were also explored in order to maximize the amount and size of single crystals produced by each reaction. Following optimization of the reactions, powder X-ray diffraction measurements (SI, Figure S2) demonstrate that the bulk products of the solvothermal reactions described here contain compounds **1**–**4** as the majority phases. There is good agreement between the lattice parameters determined from powder X-ray diffraction data (SI, Table S1) and those determined using single-crystal diffraction (Table 1). FT-IR data confirm the presence of DABCO moieties in all cases. For instance, the CH_2_ stretches centered around 2900 cm^–1^ as well as CH_2_ bends at 1400 cm^–1^ observed for DABCO are also present in compounds **1**–**4** (SI, Figure S3) and are consistent with existing literature.^25^ In compound **3**, the presence of ethanol is evidenced by a band at 3450 cm^1^, which can be attributed to the O–H stretch. A sharp band in the region 3100–3150 cm^–1^, which can be attributed to an N^+^-H stretch, is observed for compounds **3** and **4**, which contain monoprotonated (DABCOH)^+^ or diprotonated (DABCOH_2_)^2+^, respectively. Although, for protonated (DABCOH)^+^ salts, the N^+^-H stretch has been reported to give rise to a broad band between 1800 and 2800 cm^–1^, due to strong hydrogen bonding, when protonated (DABCOH)^+^ is coordinated to a transition metal, a sharp band appears at 3100 cm^–1^.^26^

### Crystal Structures

**Figure 1:**
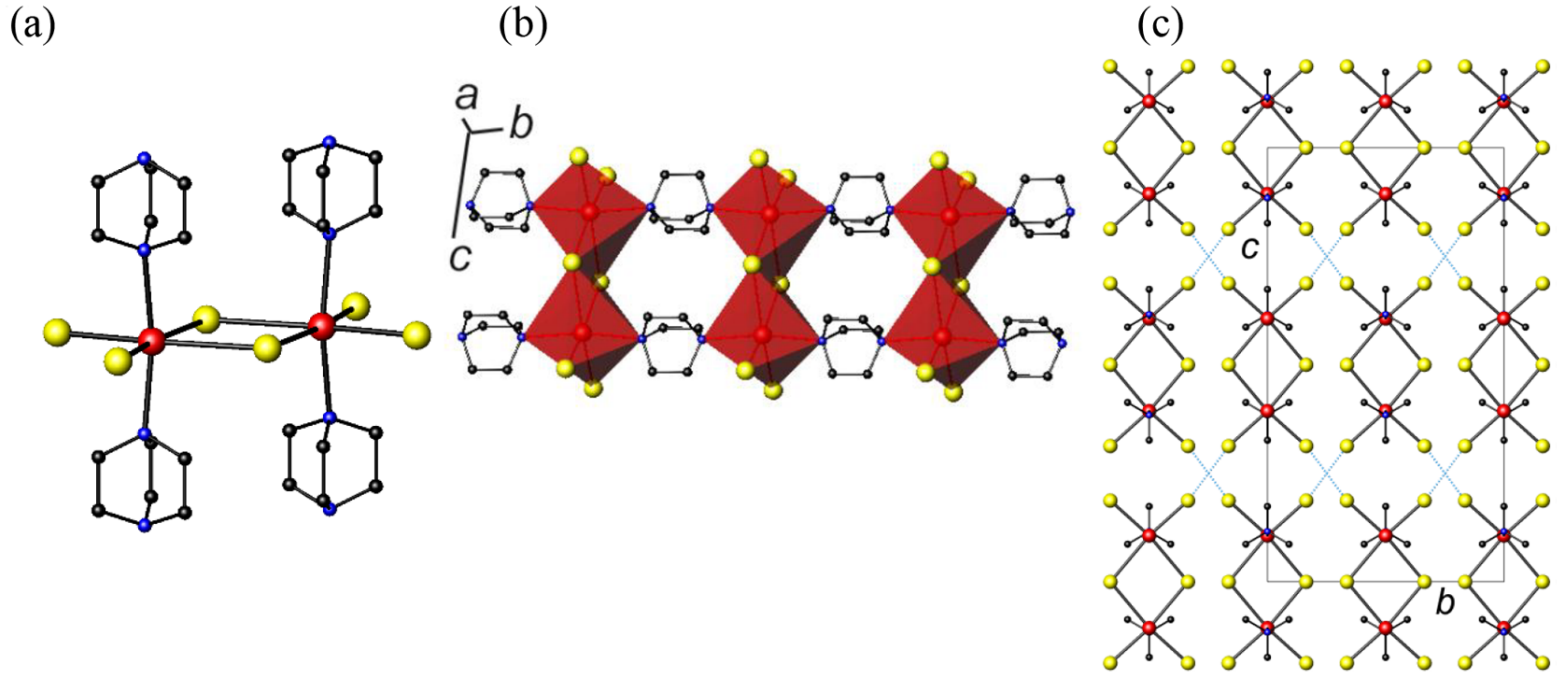
(a) View of the bismuth dimer found in **1**. (b) Polyhedral view of a (C_6_H_12_N_2_)BiI_3_ ribbon. (c) View of the crystal structure along [100]. I···I contacts are shown as dashed blue lines. Hydrogen atoms have been omitted for clarity. Key: bismuth, large red spheres; iodine, large yellow spheres; carbon, small black spheres; nitrogen, small blue spheres; bismuth dimers, red edge-sharing octahedra.

The crystal structure of (C_6_H_12_N_2_)BiI_3_ (**1**) contains one-dimensional hybrid ribbons with stoichiometry (C_6_H_12_N_2_)BiI_3_ (Figure 1). The asymmetric unit of **1** (SI, Figure S4) has only one crystallographically independent bismuth atom, which is octahedrally coordinated to four iodine atoms and two unprotonated DABCO molecules. The DABCO ligands are located in the two axial positions of the octahedron, with the halide ligands found in the four equatorial positions. The Bi–N distance of 2.613(8) Å is similar to those previously reported for organic amines coordinated to bismuth.^27^ Each (C_6_H_12_N_2_)_2_BiI_4_^–^ octahedron shares one edge with a second octahedron, forming a dimer (Figure 1a). The Bi–I distance for the μ_2_-I^–^ anion, 3.3408(6) Å, is significantly longer than that for the terminal iodide (only 2.8963(7) Å). While [Bi_2_I_10_]^4–^ anions containing two edge-sharing octahedra have been previously reported,^28^ there are very few examples of edge-sharing dimers of the type L_2_Bi_2_X_6_ (X= Cl, Br, I). When L = 2,2′-bipyridine^29^ or 1,10-phenanthroline,^30^ the organic ligands are found in equatorial positions, while when L = 4,4′-bipyridine,^31^ the arrangement is similar to that described here with the ligands found in the axial positions.

In the crystal structure of **1**, the dimers are linked into ribbons through the DABCO ligands, as illustrated in Figure 1b. These hybrid ribbons are packed parallel to the *a* axis (Figure 1c). Short I···I contacts of ca. 3.77 Å, which are significantly shorter than the sum of van der Waals’ radii for two iodine atoms (3.96 Å),^32^ link the ribbons into a pseudothree-dimensional structure. As previously discussed,^33^ this might lead to increased band dispersion and extended electronic delocalization within the crystal structure. The hybrid ribbons found in **1** contrast sharply with the crystal structures of previously reported iodobismuthates containing DABCO,^34^ which consist of discrete iodobismuthate anions, with protonated (DABCOH)^+^ and (DABCOH_2_)^2+^ acting as countercations.

**Figure 2:**
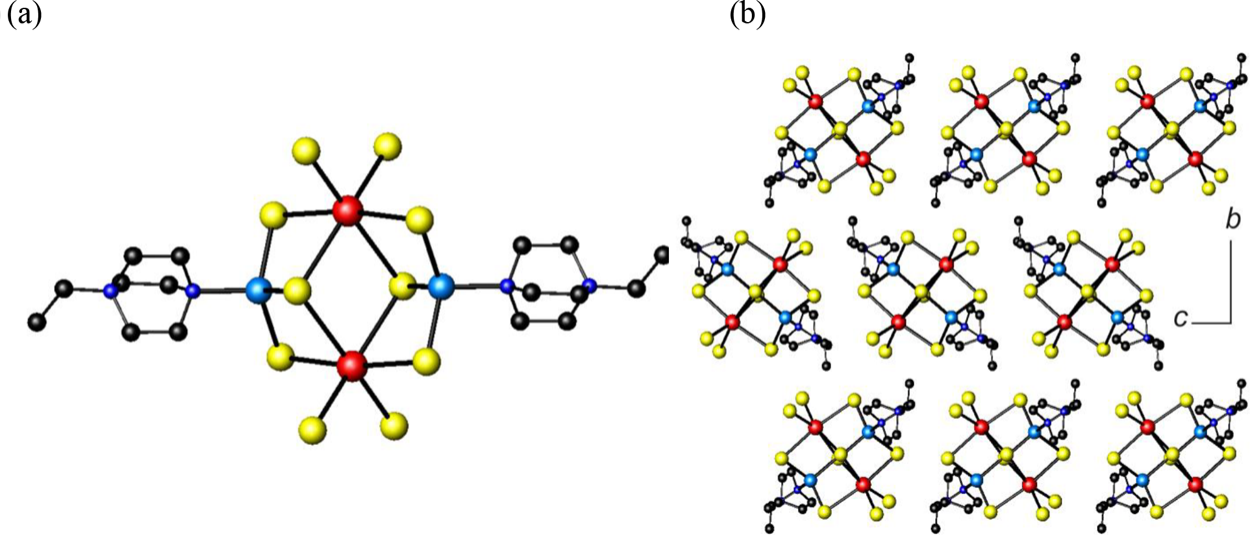
(a) The (C_8_H_17_N_2_)_2_Bi_2_Cu_2_I_10_ cluster found in **2**. (b) View of the crystal structure of **2** along [100]. Hydrogen atoms have been omitted for clarity. Key: bismuth, large red spheres; copper, large light-blue spheres; iodine, large yellow spheres; carbon, small black spheres; nitrogen, small blue spheres.

The structure of (C_8_H_17_N_2_)_2_Bi_2_Cu_2_I_10_ (**2**) consists of discrete tetranuclear clusters (Figure 2a). Within each tetranuclear cluster, two bismuth octahedra share an edge, forming a dimer. The dimer is capped by two copper tetrahedra in which each copper is coordinated to three iodine atoms and one nitrogen atom from an ethylated DABCO cation, (EtDABCO)^+^. Bond-valence sums (SI, Table S2) are consistent with Bi^3+^ and Cu^+^ oxidation states, and charge balance is therefore achieved through the ethylation of the two DABCO ligands. Although unsubstituted DABCO was added to the reaction mixture, an alkylation reaction takes place *in situ* in the presence of ethanol and DABCO, and the product of this reaction contains the 1-ethyl-1,4-diazabicyclo[2.2.2]octan-1-ium moiety. It has been previously reported that under solvothermal conditions in the presence of alcohols, DABCO can undergo alkylation reactions.^27,34^

The dinuclear bismuth unit found at the center of the cluster is similar to the frequently observed [Bi_2_I_10_]^4–^ anion,^28^ while the tetranuclear cluster itself is analogous to those previously found in [*n*-Bu_4_N][Cu_2_(CH_3_CN)_2_Bi_2_I_10_]^16b^ and [Bu_4_N]_2_[L_2_Bi_2_Cu_2_I_10_] (L = PPh_3_, P(OPh)_3_),^16d^ with acetonitrile and L replaced by ethylated DABCO cations, (EtDABCO)^+^. As is usually the case in edge-sharing iodobismuthate moieties, in **2**, the terminal Bi–I distances are shorter than the bridging Bi–I distances (average values of 2.917 and 3.195 Å, respectively). The average Cu–I distance of 2.611 Å and the Cu–N distance of 2.101(7) Å are comparable to those previously found for iodocuprates containing copper(I) coordinated to DABCO.^35^ In the crystal structure of **2**, the (C_8_H_17_N_2_)_2_Bi_2_Cu_2_I_10_ clusters are packed in layers perpendicular to the *b*-axis, forming a herringbone pattern, as shown in Figure 2b.

**Figure 3:**
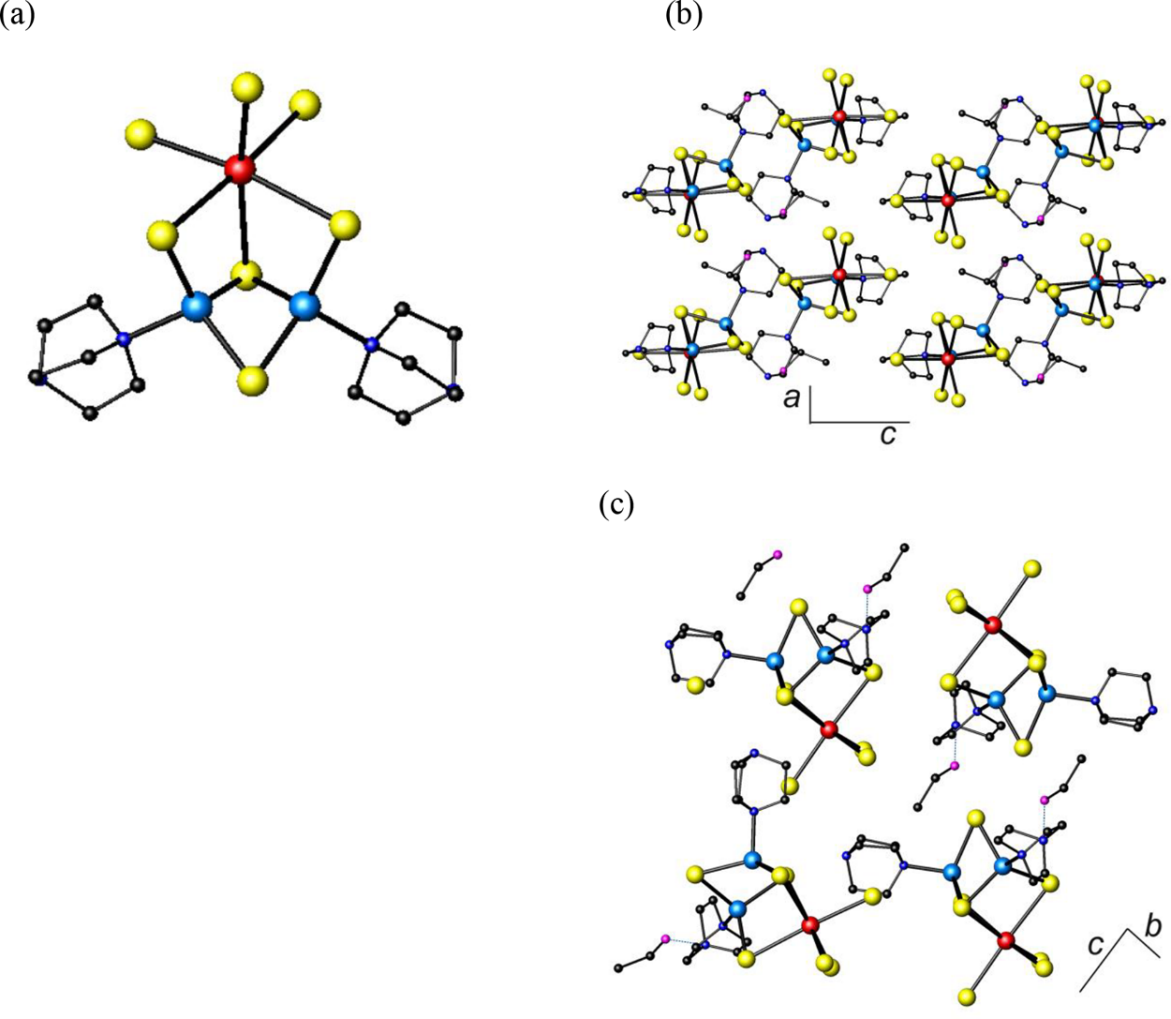
(a) The trimetallic (C_6_H_13_N_2_)_2_BiCu_2_I_7_ cluster found in **3.** (b) View of [(C_6_H_13_N_2_)_2_BiCu_2_I_7_](C_2_H_5_OH) along [010]. (c) View of [(C_6_H_13_N_2_)_2_BiCu_2_I_7_](C_2_H_5_OH) along [100], with hydrogen bonds shown as dashed blue lines. Hydrogen atoms have been omitted for clarity. Key: bismuth, large red spheres; copper, large light-blue spheres; iodine, large yellow spheres; carbon, small black spheres; nitrogen, small blue spheres; oxygen, small pink spheres.

[(C_6_H_13_N_2_)_2_BiCu_2_I_7_](C_2_H_5_OH) (**3**) contains discrete trinuclear metal clusters. There is one crystallographically independent bismuth atom, which is octahedrally coordinated by iodine, and two crystallographically independent copper atoms within the asymmetric unit (SI, Figure S6). Two of the edges of the BiI_6_ octahedron are shared with two copper tetrahedra in which each copper is coordinated to three iodine atoms and one nitrogen from a DABCO ligand (Figure 3a). A closely related trinuclear cluster coordinated to acetonitrile, [BiCu_2_I_7_(CH_3_CN)]^2–^, has been recently reported.^16^^c^ While in **3**, both copper atoms are tetrahedrally coordinated, in [BiCu_2_I_7_(CH_3_CN)]^2–^, one of the copper atoms exhibits only trigonal pyramidal coordination, as it is not bonded to an acetonitrile ligand.

Bond-valence sums (SI, Table S3) for **3** are consistent with trivalent and monovalent oxidation states for bismuth and copper, respectively. To achieve charge balance, the two DABCO ligands coordinated to copper must be therefore monoprotonated, (DABCOH)^+^. A number of iodocuprates containing copper coordinated to (DABCOH)^+^ are known, as exemplified by [Cu_4_I_5_(DABCOH^+^)CH_3_CN] and [Cu_7_I_8_(DABCOH^+^)DABCO].^35b^ While the Cu–Bi distances found in **3** are similar to those in **2**, the Cu–Cu distance, which is 2.669(3) Å, is close to the interatomic distance of 2.56 Å found in copper metal^36^ and below the sum of the van der Waals’ radii (2.8 Å) of two copper atoms. It is known that metallophilic *d*^10^–*d*^10^ interactions in copper(I) compounds, reflected by short copper–copper distances lying between 2.34 and 2.79 Å, often influence the structure of these compounds.^37^

**Figure 4:**
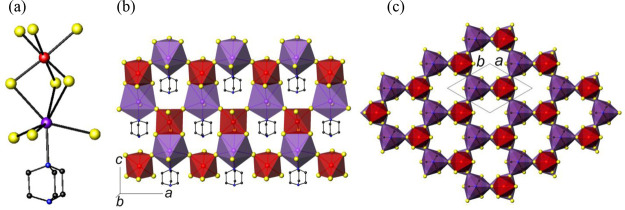
(a) The (DABCO)KBiI_9_^5–^ building block found in **4**. (b) Polyhedral view of a corrugated sheet formed by linkage of (DABCO)KBiI_9_^5–^ building blocks. (c) Polyhedral view of the crystal structure of **4** along [001]. Hydrogen atoms have been omitted for clarity. Key: bismuth, large red spheres; iodine, large yellow spheres; potassium, large purple spheres; carbon, small black spheres; nitrogen, small blue spheres; bismuth octahedra, red; potassium capped octahedra, purple.

In **3**, the trinuclear clusters are packed in columns parallel to the *b*-axis, as shown in Figure 3b. Ethanol, which was used as the solvent in this reaction, is incorporated into the final crystal structure. The oxygen in the ethanol molecule has one nitrogen neighbor in a DABCO molecule at ca. 2.83 Å, implying hydrogen-bonding interactions between the solvent molecules and the trinuclear clusters (Figure 3c).

(C_6_H_14_N_2_)[(C_6_H_12_N_2_)KBiI_6_] (**4**) is a rare example of a 3-dimensional iodobismuthate. The building block of **4** is a dinuclear unit formed by face sharing of a BiI_6_ octahedron and a KI_6_(DABCO) capped octahedron (Figure 4a). The Bi–I bond lengths of 3.028(1) and 3.124(2) Å are slightly longer than those found in **1**, **2**, and **3** for terminal I^–^ but lie within the range expected for bridging μ_2_-I^–^ ligands. The K–N distance, 3.00(2) Å, is comparable to that found in K(NH_2_), 3.065 Å.^38^ There are three short K–I distances, with a value of 3.539(3) Å and three longer K–I distances, of 3.739(6) Å, indicating that the coordination environment around the potassium is highly distorted. These bond lengths are similar to those in KBiI_4_·4H_2_O,^39^ where potassium is coordinated to six iodine atoms and two water molecules.

In the crystal structure of **4**, the dinuclear units, (DABCO)KBiI_9_^5–^, are linked by four of the six terminal iodines to four other dinuclear units to form corrugated sheets (Figure 4b), which are oriented parallel to the *ac* plane. Linkage between sheets by the two remaining terminal iodines results in the formation of a 3-dimensional structure (Figure 4c), which contains hexagonal channels of *ca*. 6 Å diameter (when measured from iodine to iodine) oriented along the *c*-axis. This corresponds to approximately 28% of void space. Bond-valence sums (SI, Table S4) indicate that bismuth is in the trivalent state, and therefore the crystallographically determined formula of [(C_6_H_12_N_2_)KBiI_6_]^2–^ does not charge balance. Elemental analysis is consistent with the presence of disordered diprotonated (DABCOH_2_)^2+^ cations within these channels, resulting in the final formula of (C_6_H_14_N_2_)[(C_6_H_12_N_2_)KBiI_6_] (or (DABCOH_2_)[(DABCO)KBiI_6_]). Only a few potassium iodobismuthates have been previously reported: K_3_Bi_2_I_9_, which contains corrugated layers of corner-connected BiI_6_ octahedra,^12^^(b)^ KBiI_4_·4H_2_O, which contains one-dimensional [BiI_4_]^−^ chains,^39^ ([4-NCMePy]_5_[K(BiI_6_)_2_]), containing trinuclear discrete anions [K(BiI_6_)_2_]^5–^,^40^ and K_18_Bi_8_I_42_(I_2_)_0.5_·14H_2_O, which consists of corner-sharing and edge-sharing pairs of BiI_6_ octahedra.^41^ The compound reported here therefore constitutes the first three-dimensional example.

### Thermal Stability

Thermogravimetric data (SI) indicate that under a nitrogen atmosphere, compounds **1**, **2**, and **4** are stable up to approximately 300 °C, while **3** is stable up to 220 °C. For **1**, **2**, and **4**, which decompose in two steps, the first decomposition step corresponds to the loss of the DABCO moieties as well as of iodine. The second step, which occurs at much higher temperatures, above 500–600 °C, seems to correspond to bismuth starting to volatilize. In the case of **3**, the first decomposition step, which starts at 220 °C, corresponds to the loss of ethanol and is followed by a second step at 280 °C during which DABCO and iodine are lost.

### UV–vis Diffuse Reflectance and Photoluminescence

**Figure 5:**
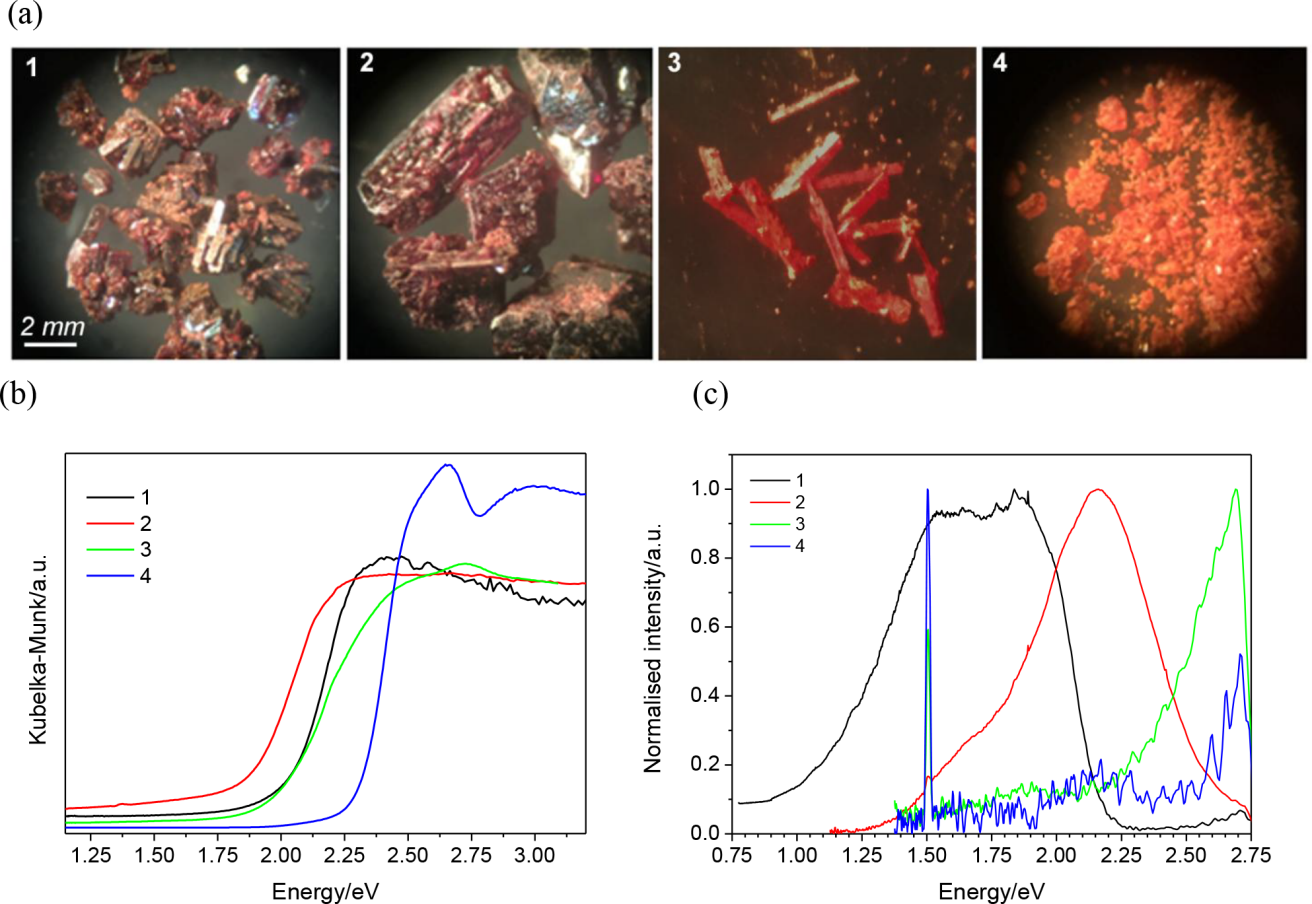
(a) Photographs of crystals of reactions producing compounds **1**–**4**. (b) UV–vis diffuse reflectance data for compounds **1** (black), **2** (red), **3** (green), **4** (blue). (c) Photoluminescence spectra of compounds **1** (black), **2** (red), **3** (green), **4** (blue). The sharp feature at ∼1.5 eV is the second order grating diffraction of residual scattered laser excitation.

As illustrated in Figure 5a, all the compounds consist of well-formed red/orange crystals. The UV–vis diffuse reflectance data collected on ground crystals of **1**–**4**, which are shown in Figure 5b, are consistent with the color of the crystals. The optical band gaps, which were estimated from the absorption edge,^42^ exhibit values of 1.96(6), 1.82(5), 1.91(5), and 2.27(8) eV for **1**–**4** respectively. Tauc plots^43^ with exponents of n = 1/2 (direct allowed transition) and n = 2 (indirect allowed transition) resulted in fits of very similar quality, hence it was not possible to identify the nature of the transition in each case. Although the band gaps for these materials, which are blue-shifted with respect to that of condensed BiI_3_ (1.67 eV),^44^ are higher than the ideal value given by the Shockley–Quiesser limit for single-junction solar cells,^45^ these materials may be suitable for multijunction solar cells. The peak found for compound **4** at 2.65 eV might be attributed to an exciton,^46^ and given that this is observable at room temperature, the binding energy for the exciton may be relatively large.

To provide further insight into the optical properties of these materials, photoluminescence studies were undertaken. As shown in Figure 5c, compounds **1** and **2** both displayed broad luminescence centered at ∼1.68 eV (740 nm) and ∼2.16 eV (575 nm), respectively. For compounds **3** and **4**, only a weak partial spectrum could be measured, and we therefore await future low-temperature studies to confirm any assignment of the recorded emission to the compounds. Compound **1** displays a large (∼280 meV) shift between the photoluminescence maximum and the estimated optical band gap, indicative of a strong exciton binding energy. For compound **2**, we note that there is a sizable overlap of the measured optical absorption and the photoluminescence, with the photoluminescence peak being ∼0.34 eV higher in energy than the estimated value of the optical band gap. This may indicate that a broad excitonic absorption is convolved with that of the optical band gap absorption.^47^ Exciton binding energies of ∼300 meV have previously been reported for bismuth halides,^48^ hence such a shift is not unreasonable. We note that emission displayed by [Dim]_2_[Bi_2_I_10_] (Dim^2+^ = C_9_H_14_N_4_^2+^) also presents an emission peak (∼2.0 eV) above the reported optical bandgap (1.9 eV).^49^ Compound **2** displayed the brightest emission though the quantum yield remained too low to allow for accurate measurement. A low-temperature (5.5 K) measurement of the photoluminescence of **2** (SI, Figure S9) showed a blueshift in the emission spectrum with decreasing temperature, combined with the loss of the shoulder observed within the low-energy tail at 300 K. We finally note that the lack of a distinct maximum for the emission observed from **1** was also reproducible indicating a degree of instability of the intensity. However, we did not notice any overall reduction in the overall signal strength during remeasurement and believe that sample to be photostable. The reflectance data for this compound also displays similar noise-like features at high energy. A comprehensive study of the optical properties is the subject of current work and will be reported in due course.

The major factor that determines the optical properties of hybrid iodobismuthates is the inorganic moieities, because the main contributor to the top of the valence band are iodine 5*p* states, while the bottom of the conduction band is primarily formed by Bi 6*p* states.^33^ Alkali-metal cations do not contribute in a significant way to the valence band,^39^ while Cu^+^ cations have been found to reduce the band gap by introducing states at the top of the valence band.^16a^ It has been also been shown that the optical band gap decreases on increasing the dimensionality of the inorganic anion,^50^ which can augment the degree of band dispersion.^41^ In addition to the nature of the inorganic anion and its dimensionality, iodide–iodide interactions or the orientation and separation of the anions can also have a noticeable influence on the magnitude of the band gap.^46,34,33a^ All of these factors are likely to contribute to the experimental values reported here.

### Thin-Film Deposition

**Figure 6:**
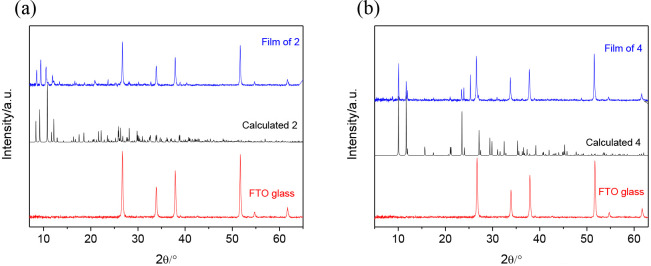
Powder diffraction pattern of a film of (a) compound **2** and (b) compound **4**, drop-coated onto FTO glass (blue line). The pattern of FTO glass (red line) and the calculated pattern of compounds **2** and **4** (black line) are shown for comparison purposes.

While compound **1** is not soluble in acetonitrile and only sparingly soluble in DMF, compounds **2**, **3**, and **4** dissolve in both acetonitrile and DMF, producing colored solutions (SI, Figure S10). DMF solutions of **2** and **4** have been successfully used for the preparation of thin films by drop-casting, as illustrated in Figure 6.

For insoluble compounds, which cannot be deposited from a solution, spin coating from suspensions is a viable alternative. This has been demonstrated for the construction of solar cells, using SnS^51^ or Cu(In,Ga)Se_2_,^52^ and requires the optimization of the suspension used for spin coating by exploring the use of ultrasonication or wet milling to improve the dispersion of the solid into the suspension. This work is beyond the scope of this paper.

## Discussion

Solvothermal synthesis has been previously exploited for the discovery of new iodobismuthates,^33a,34,46,53^ as growth of single crystals is normally required for structural characterization. The products of such reactions depend on the subtle interplay of a wide range of variables, including the composition of the reaction mixtures, pH, pressure, and temperature. Solvothermally prepared iodobismuthates, which are often synthesized in the presence of hydriodic acid, usually consist of iodobismuthate anions and organic cations.^6^ Reducing the pH of the reaction mixture, through the addition of HI, leads to the protonation of the organic amines present in the reaction mixture. For instance, in aqueous solutions at ambient conditions, monoprotonated (DABCOH)^+^ is the predominant species for pH < 8, while diprotonated (DABCOH_2_)^2+^ is the predominant moiety in solution for pH < 3.^25^ In previously reported iodobismuthates, DABCO was incorporated as a countercation, either as a protonated or an alkylated species.^27,34,54^ Here, we have shown that by carrying reactions in the absence of hydriodic acid, DABCO can also act as a linker between inorganic building blocks, as exemplified by compound **1**. Moreover, we have found that the use of alcohols as solvents can result in undesirable alkylation reactions. Therefore, in order to exploit DABCO as a ditopic linker in a systematic manner, the identification of alternative solvents, such as ethylene glycol which was used for the synthesis of **1**, will be essential. Attempts to use acetonitrile or 2-butanone, either as pure solvents or as mixtures with ethanol, have so far failed to produce good-quality single crystals suitable for structural characterization. For reaction products containing other metal cations, our results indicate that in the presence of Bi^3+^ and Cu^+^, DABCO preferentially coordinates to copper. In iodocuprates containing DABCO, this amine frequently acts as a ligand,^35^ suggesting that under solvothermal conditions, the coordination abilities of DABCO and I^–^ toward Cu^+^ are very similar. The preferential coordination of DABCO to K^+^ found in compound **4** may be justified by the Hard and Soft Acid and Base (HSAB) principle.

As is often the case in solvothermal synthesis, the reactions described here appear to involve redox processes, as evidenced by the presence of bismuth metal in the product of the unoptimized reaction for compound **2** (SI, Figure S1b). While the metal cations in the compounds **1**–**4** remain in the same oxidation states as those in the reagents, the unidentified impurities may consist of redox products. Under solvothermal conditions, amines and ethylene glycol can act as weak reducing agents, and organic species often undergo decomposition reactions. Further optimization of the reaction conditions may produce pure phases; here, we sought to produce large amounts of single crystals for complete characterization. Alternatively, scale up of the reactions described here, using larger 125 mL autoclaves, could be followed by purification, by washing the impurities with a suitable solvent in which the target compound is not soluble. This approach has been successfully implemented by Mitzi and Brock for the purification of (H_2_DDDA)BiI_5_ crystals.^46^

Although it has been suggested that the heterovalent substitution of Pb^2+^ by Bi^3+^ and Cu^+^ may result in the formation of double perovskites, a recent high-throughput computational study indicates that double perovskites, such as Cs_2_CuSbBr_6_, Cs_2_CuBiCl_6_, and Cs_2_CuBiBr_6_, which had been previously predicted to be stable,^55^ are in fact thermodynamically unstable.^56^ The smaller ionic radius of Cu^+^ (0.77 Å) when compared to those of Ag^+^ or Bi^3+^ (1.15 and 1.03 Å respectively)^57^ favors tetrahedral coordination for Cu^+^, as shown by theoretical calculations^58^ and as exemplified by compounds **2** and **3** and the condensed phase CuBiI_4_.^59^ It should be noted that although copper is described as octahedrally coordinated in the recently reported [CuBiI_8_]^4–^ layers, the pseudo-octahedral copper environment found in these layers is highly distorted, with two unusually long Cu–I distances of 3.793 and 4.048 Å.^18a^ The number of known copper iodobismuthates is still rather small,^16−18^ and the discrete tetranuclear and trinuclear clusters found here may become building blocks for hybrid materials with extended structures. Closely related tetranuclear clusters containing Ag have indeed been found in the one-dimensional chains [Bi_2_Ag_2_I_10_^2–^]_n_.^50^

## Conclusions

In summary, we have shown that in solvothermal reactions carried out in the absence of hydriodic acid, DABCO can act as a ligand instead of a countercation. This provides a route for the synthesis of iodobismuthate coordination polymers, as exemplified by compound **1**. Inclusion of Cu^+^ in the reaction mixtures does not lead to the formation of double perovskites, as the relatively small radius of Cu^+^ appears to favor tetrahedral rather than octahedral coordination. However, the tetranuclear and trinuclear clusters found in **2** and **3** could be used as building blocks for copper iodobismuthate coordination polymers, and this would open an alternative route to control dimensionality. Given that most binary iodobismuthates are either discrete anions or one-dimensional chains, and that higher dimensionalities are desirable to reduce the band gap and increase the band dispersion, the three-dimensional structure of compound **4** is particularly promising. Further exploratory synthesis of ternary iodobismuthates containing alkali metals and, in particular, exploration of the effect of the alkali metal/bismuth ratio on the structure and properties might lead to materials with properties comparable to those of MAPI.

## Supporting Information Available

Powder X-ray diffraction, TGA, and FTIR data for compounds **1**–**4**; asymmetric units for compounds **1**–**4**, bond-valence sums for compounds **2**–**4**, and low-temperature photoluminescence spectrum for compound **2** (PDF)

## Accession Codes

CCDC 2020552–2020555 contain the supplementary crystallographic data for this paper. These data can be obtained free of charge via www.ccdc.cam.ac.uk/data_request/cif, or by emailing data_request@ccdc.cam.ac.uk, or by contacting The Cambridge Crystallographic Data Centre, 12 Union Road, Cambridge CB2 1EZ, UK; fax: +44 1223 336033.
