# Supplementary material for: Multiple Roles of 1,4-Diazabicyclo[2.2.2]octane in the Solvothermal Synthesis of Iodobismuthates
Source: Inorg Chem. 2021 Mar 22;60(7):5333–42. doi: 10.1021/acs.inorgchem.1c00318 (PMC8041286; doi:10.1021/acs.inorgchem.1c00318)
Supplement: Supplementary file 1 — ic1c00318_si_001.pdf [file ic1c00318_si_001.pdf]

Supporting Information

# Multiple roles of 1,4-diazabicyclo[2.2.2]octane in the solvothermal synthesis of iodobismuthates

*Yunhe Cai,[1] Ann M. Chippindale,[1] Richard J. Curry[2] and Paz Vaqueiro[1*]*

[1]Department of Chemistry, University of Reading, Whiteknights, Reading, Berkshire, RG6 6DX, United Kingdom.

[2]Photon Science Institute, Department of Electrical and Electronic Engineering, University of Manchester, Manchester, M13 9PL, United Kingdom.

Corresponding author E-mail: p.vaqueiro@reading.ac.uk

**Initial (unoptimised) syntheses:**

**Compound 1:** Ethylene glycol (10 ml) was added to $BiI_3$ (0.5923g, 1 mmol), CuI (0.0315g, 0.2 mmol) and DABCO (0.1109g, 1 mmol) with stirring. The mixture was sealed into a Teflon-lined stainless steel autoclave, heated at 140ºC for a period of 5 days and then cooled slowly to room temperature at 1 ° C $min^{-1}$. The product was a mixture of an orange powder, together with red crystals of compound **1** and a small amount of crystals of compound **3**.

**Compound 2:** Ethanol (10 ml) was added to $BiI_3$ (0.5887 g, 1 mmol), CuI (0.0321 g, 0.2 mmol) and DABCO (0.1122g, 1 mmol). The mixture was sealed into a Teflon-lined stainless steel autoclave, heated at 170ºC for a period of 5 days and then cooled slowly to room temperature at 0.2° C $min^{-1}$. The product consisted of a mixture of red powder and crystals, together with bismuth metal.

**Compound 3:** Ethanol (10 ml) was added to $BiI_3$(0.5840 g, 1 mmol), CuI (0.1986 g, 1 mmol), KI (0.5014 g, 3 mmols) and DABCO (0.1161 g, 1 mmol). The mixture was sealed into a Teflon-lined stainless steel autoclave, heated at 140ºC for a period of 5 days and then cooled slowly to room temperature at 0.2° C $min^{-1}$. The product consisted mainly of a red powder, together with a small amount of red crystals.

**Compound 4**: Ethanol (10ml) was added to $BiI_3$ (0.5840g, 1mmol), KI (0.1170 g, 0.7 mmol) and DABCO (0.1161g, 1 mmol). The mixture was sealed into a Teflon-lined stainless steel autoclave, heated at 140ºC for a period of 5 days and then cooled slowly to room temperature at 0.2° C $min^{-1}$. The product was a mixture of red powder and orange crystals.

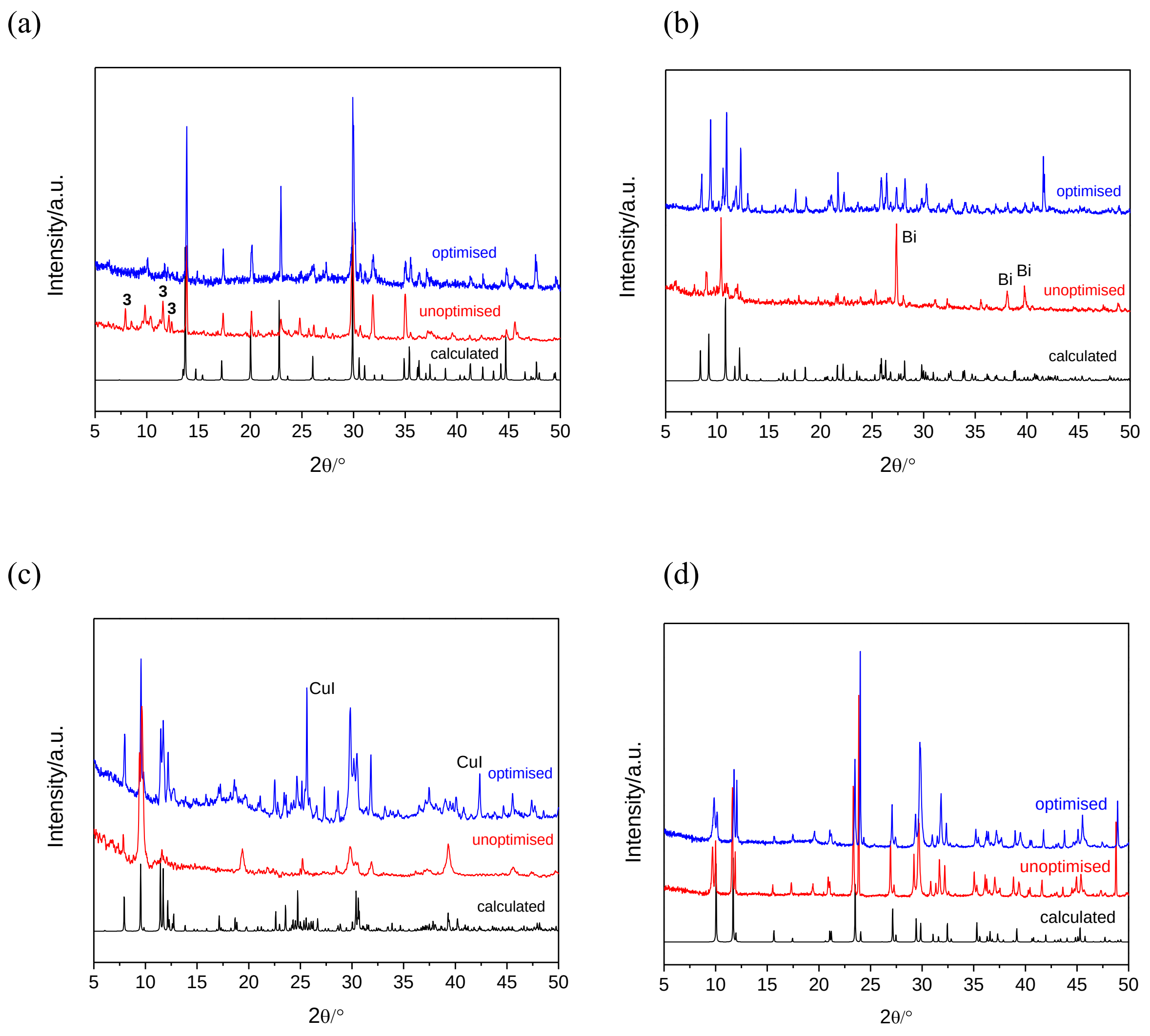


**Figure S1.** Experimental powder X-ray diffraction patterns collected for bulk products of the unoptimised reactions (red) and optimised reactions (blue) producing compounds (a) **1**, (b) **2**, (c) **3**, (d) **4**. The patterns calculated using the structures determined by single-crystal X-ray diffraction are shown in black. Identified impurities are labeled.

**Table S1.** Lattice parameters of compounds **1-4** determined at room temperature using powder X-ray diffraction data.

| Compound | **1** | **2** | **3** | **4** |
|---|---|---|---|---|
| *a*/Å | 7.8082(14) | 9.0149(24) | 8.964(12) | 10.2404(26) |
| *b*/Å | 13.1822(23) | 21.2925(54) | 11.962(20) | 10.2404(26) |
| *c*/Å | 24.1004(29) | 10.8990(27) | 29.213(41) | 14.9329(37) |
| *β*/° | 90 | 96.521(11) | 91.522(91) | 90 |

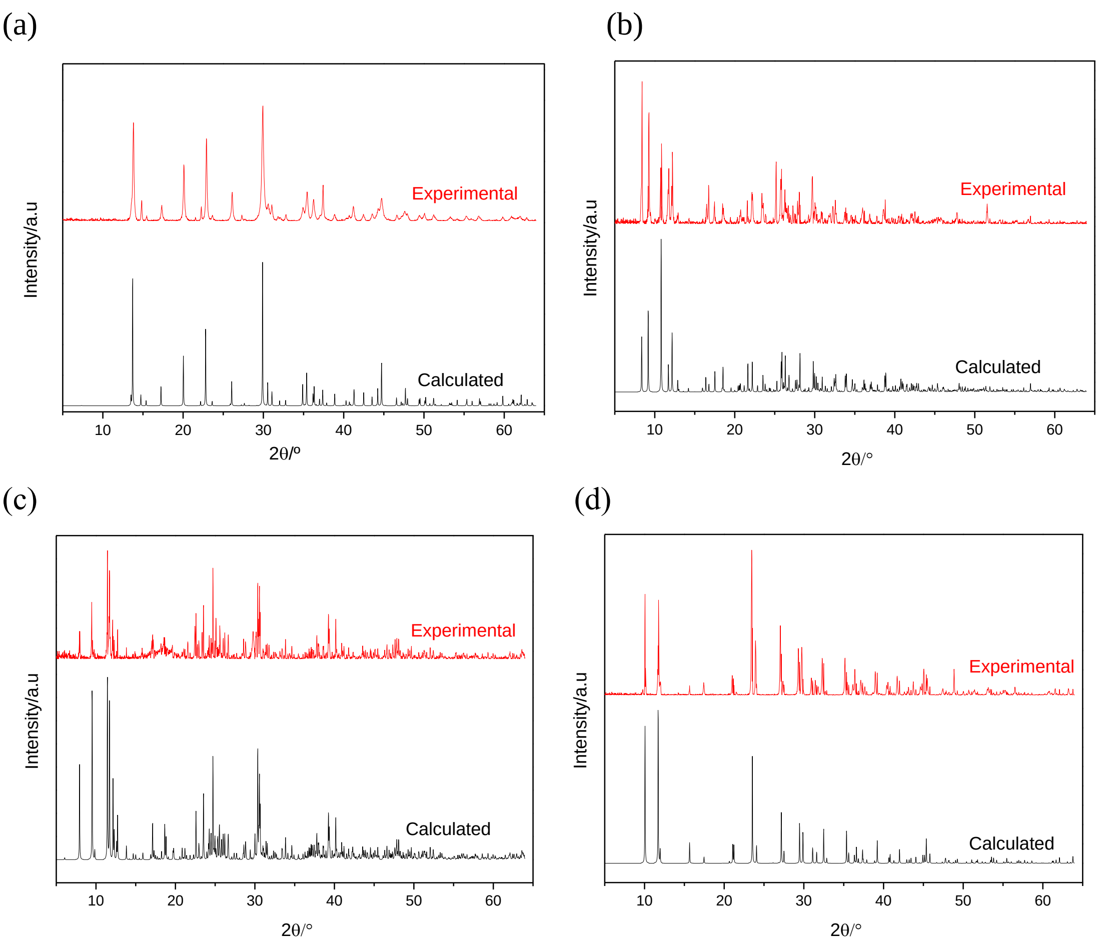


**Figure S2.** Experimental powder X-ray diffraction patterns (red) collected for handpicked crystals of the reactions producing (a) **1**, (b) **2**, (c) **3**, (d) **4**. The patterns calculated using the structures determined by single-crystal X-ray diffraction are shown in black.

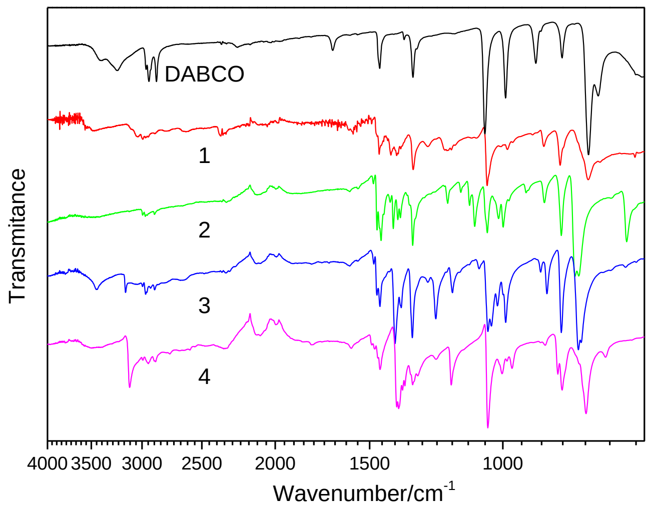


**Figure S3.** FT-IR spectra for hand-picked crystals of compounds **1-4** and DABCO.

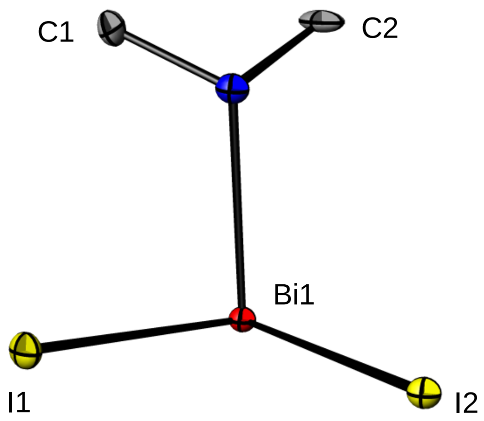


**Figure S4.** Asymmetric unit of compound **1**. Hydrogen atoms have been omitted for clarity.

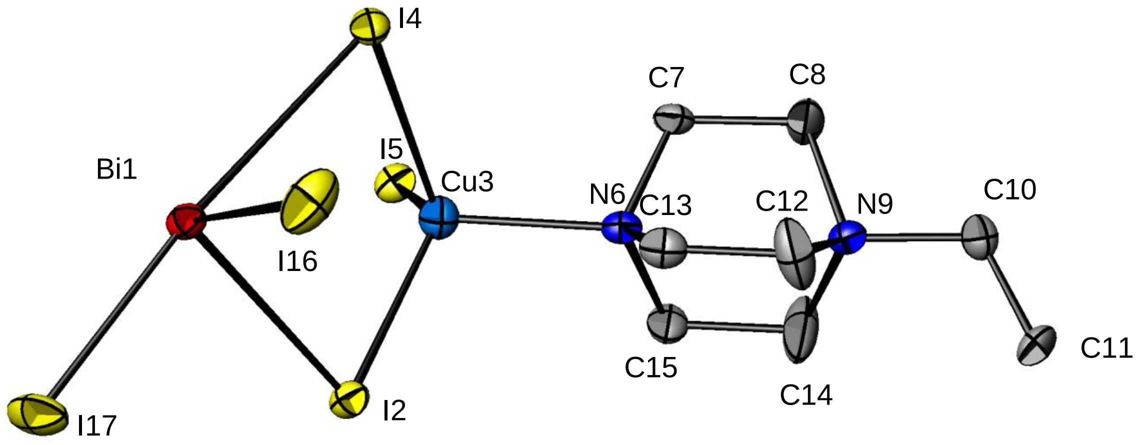


**Figure S5.** Asymmetric unit of compound **2**. Hydrogen atoms have been omitted for clarity.

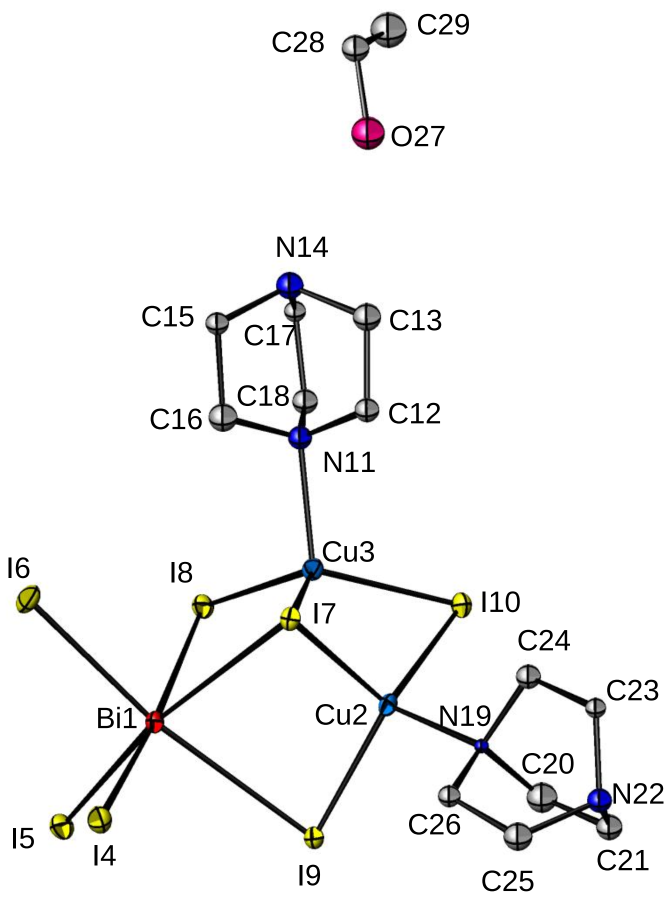


**Figure S6.** Asymmetric unit of compound **3**. Hydrogen atoms have been omitted for clarity.

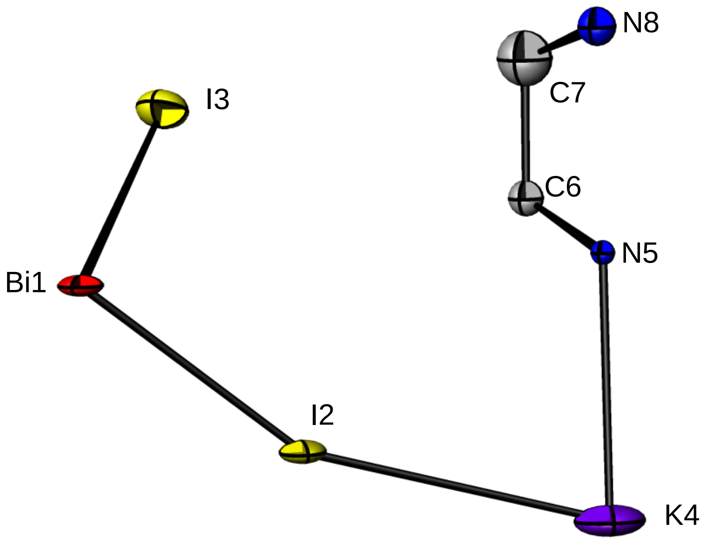


**Figure S7.** Asymmetric unit of compound **4**. Hydrogen atoms have been omitted for clarity.

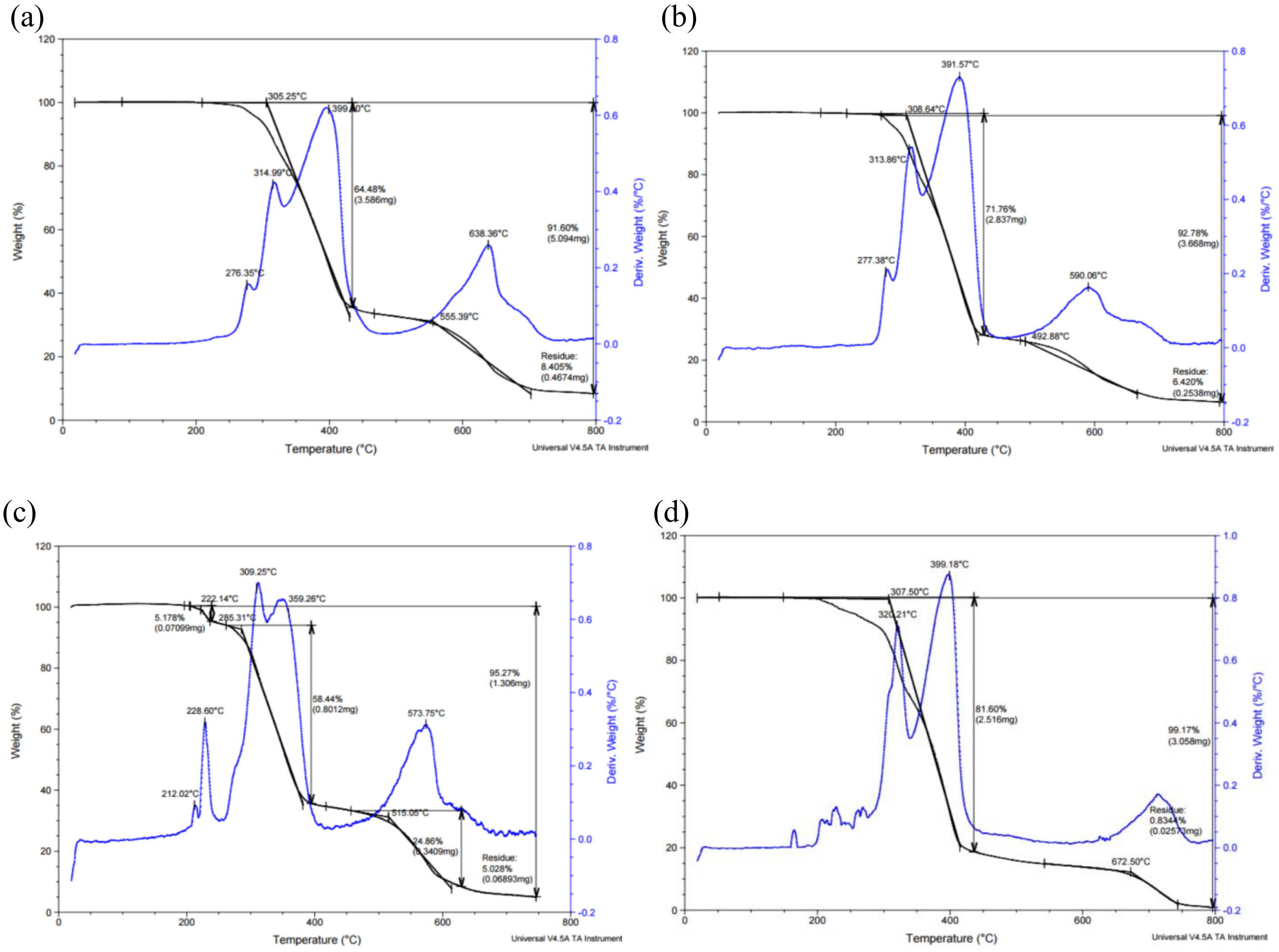


**Figure S8.** Thermogravimetric data collected for handpicked crystals of compounds (a) **1**, (b) **2**, (c) **3**, (d) **4.**

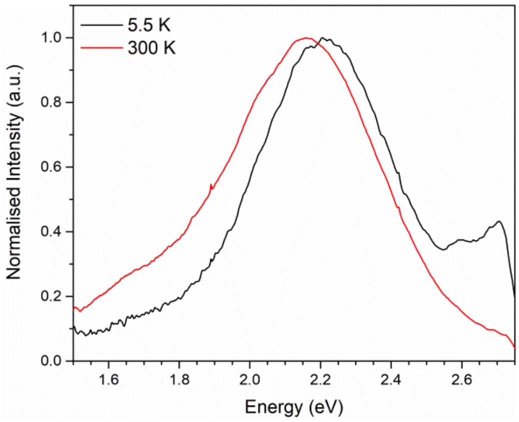


**Figure S9.** 405 nm excited photoluminescence spectrum of compound **2** collected at 5.5 K (black) and 300 K (red). The increase in signal strength above ~2.5 eV in the 5.5 K spectrum is due to the detection of background emission from the excitation source.

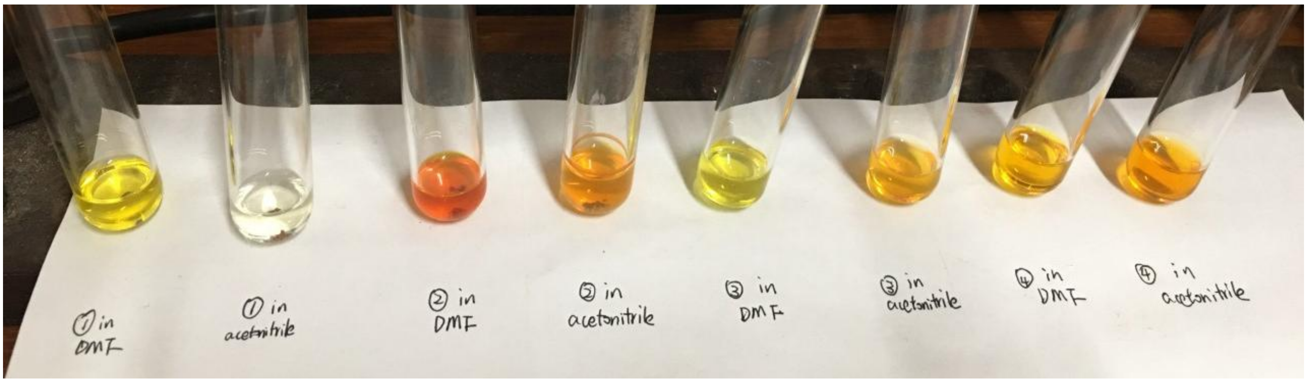


**Figure S10.** Photograph of the solubility tests of compounds **1-4** in acetonitrile and DMF at room temperature.

**Table S2.** Bond-valence sums for compound **2**.

| | | d/Å | v |
|---|---|---|---|
| Bi | I | 3.2842 | 0.285 |
| | | 3.1039 | 0.464 |
| | | 2.9369 | 0.729 |
| | | 3.3315 | 0.251 |
| | | 3.0613 | 0.521 |
| | | 2.898 | 0.810 |
| | | Total | 3.060 |

| | | d/Å | v |
|---|---|---|---|
| Cu | I | 2.5845 | 0.313 |
| | | 2.6396 | 0.270 |
| | | 2.6085 | 0.294 |
| | N | 2.107 | 0.275 |
| | | Total | 1.152 |

**Table S3.** Bond-valence sums for compound **3**.

| | | d/Å | v |
|---|---|---|---|
| Bi | I | 3.324 | 0.256 |
| | | 3.008 | 0.602 |
| | | 2.927 | 0.749 |
| | | 2.932 | 0.740 |
| | | 3.172 | 0.386 |
| | | 3.261 | 0.304 |
| | | Total | 3.037 |

| | | d/Å | v |
|---|---|---|---|
| Cu2 | I | 2.658 | 0.257 |
| | | 2.610 | 0.292 |
| | | 2.612 | 0.291 |
| | N | 2.115 | 0.270 |
| | | Total | 1.110 |

| | | d/Å | v |
|---|---|---|---|
| Cu3 | I | 2.632 | 0.275 |
| | | 2.599 | 0.301 |
| | | 2.609 | 0.293 |
| | N | 2.162 | 0.237 |
| | | Total | 1.107 |

**Table S4.** Bond-valence sums for compound **4**.

| | | d/Å | v |
|---|---|---|---|
| Bi | I | 3.1235 | 0.440 |
| | | 3.1235 | 0.440 |
| | | 3.1235 | 0.440 |
| | | 3.0278 | 0.570 |
| | | 3.0278 | 0.570 |
| | | 3.0278 | 0.570 |
| | | Total | 3.032 |

| | | d/Å | v |
|---|---|---|---|
| K | I | 3.539 | 0.168 |
| | | 3.539 | 0.168 |
| | | 3.539 | 0.168 |
| | | 3.739 | 0.098 |
| | | 3.739 | 0.098 |
| | | 3.739 | 0.098 |
| | N | 3.000 | 0.135 |
| | | Total | 0.935 |
